# Dihydroartemisinin-Piperaquine Treatment of Multidrug Resistant Falciparum and Vivax Malaria in Pregnancy

**DOI:** 10.1371/journal.pone.0084976

**Published:** 2014-01-17

**Authors:** Jeanne Rini Poespoprodjo, Wendy Fobia, Enny Kenangalem, Daniel A. Lampah, Paulus Sugiarto, Emiliana Tjitra, Nicholas M. Anstey, Ric N. Price

**Affiliations:** 1 District Health Authority, Timika, Papua, Indonesia; 2 Menzies School of Health Research-National Institute of Health Research and Development Research Program, Timika, Papua, Indonesia; 3 Mitra Masyarakat Hospital, Timika, Papua, Indonesia; 4 National Institute of Health Research and Development, Ministry of Health, Jakarta, Indonesia; 5 Global Health Division, Menzies School of Health Research and Charles Darwin University, Darwin, Northern Territory, Australia; 6 Division of Medicine, Royal Darwin Hospital, Darwin, Northern Territory, Australia; 7 Centre for Tropical Medicine, Nuffield Department of Clinical Medicine, University of Oxford, Oxford, United Kingdom; University of Barcelona, Spain

## Abstract

**Background:**

Artemisinin combination therapy (ACT) is recommended for the treatment of multidrug resistant malaria in the second and third trimesters of pregnancy, but the experience with ACTs is limited. We review the exposure of pregnant women to the combination dihydroartemisinin-piperaquine over a 6 year period.

**Methods:**

From April 2004–June 2009, a prospective hospital-based surveillance screened all pregnant women for malaria and documented maternal and neonatal outcomes.

**Results:**

Data were available on 6519 pregnant women admitted to hospital; 332 (5.1%) women presented in the first trimester, 324 (5.0%) in the second, 5843 (89.6%) in the third, and in 20 women the trimester was undocumented. Peripheral parasitaemia was confirmed in 1682 women, of whom 106 (6.3%) had severe malaria. Of the 1217 women admitted with malaria in the second and third trimesters without an impending adverse outcome, those treated with DHP were more likely to be discharged with an ongoing pregnancy compared to those treated with a non-ACT regimen (Odds Ratio OR = 2.48 [1.26–4.86]); p = 0.006. However in the first trimester 63% (5/8) of women treated with oral DHP miscarried compared to 2.6% (1/38) of those receiving oral quinine; p<0.001. Of the 847 women admitted for delivery those reporting a history of malaria during their pregnancy who had been treated with quinine-based regimens rather than DHP had a higher risk of malaria at delivery (adjusted OR = 1.56 (95%CI 0.97–2.5), p = 0.068) and perinatal mortality (adjusted OR = 3.17 [95%CI: 1.17–8.60]; p = 0.023).

**Conclusions:**

In the second and third trimesters of pregnancy, a three day course of DHP simplified antimalarial treatment and had significant benefits over quinine-based regimens in reducing recurrent malaria and poor fetal outcome. These data provide reassuring evidence for the rational design of prospective randomized clinical trials and pharmacokinetic studies.

## Introduction

Malaria in pregnancy (MiP) is an major global health problem associated with an increased risk of severe adverse events contributing to both maternal and infant mortality [1], [2]. Although early diagnosis and prompt treatment with effective antimalarial drugs are important interventions for reducing these adverse outcomes [3], [4], however this strategy is under threat from the emergence and spread of multidrug resistant parasites [5]. The World Health Organisation advocates three alternatives for the treatment of malaria in the second and third trimesters of pregnancy: an ACT known to be effective in the country/region, a 7 day course of artesunate plus clindamycin, or a 7 days course of quinine plus clindamycin. In view of the lack of safety data WHO guidelines do not recommended the use of ACTs in the first trimester [6]. However recent evidence from clinical trials has highlighted the adverse impact of even a single episode of malaria in the first trimester of pregnancy and its association with miscarriage [7]. These observations have lead some authorities to call for a review of the safety and efficacy of artemisinin based treatment regimens with regard to optimizing malaria the management in early as well as late pregnancy. There is a clear need for highly effective antimalarial treatments that can applied safely in early pregnancy.

Experience with the use of ACTs in pregnancy is accruing, although evidence of the public health impact of this policy is limited [8]. Despite this in most malaria endemic regions antimalarial treatment of pregnant women remains reliant on quinine, chloroquine or sulfadoxine-pyrimethamine (SP) based regimens. However the use of these conventional treatments become increasingly compromised by the widespread drug resistance to chloroquine and SP [9], [10].

In Papua, Indonesia, high grade resistance to chloroquine (CQ) and sulfadoxine-pyrimethamine (SP) is prevalent in both *P. falciparum* and *P. vivax* [11], and few options exist for the treatment of MiP. Historically the alternative treatment has been a 7 day course of unsupervised quinine, but evidence suggests that this is rarely adhered to and when prescribed as an unsupervised treatment its effectiveness falls to less than 45% [11]. In this region previous clinical trials have demonstrated dihydroartemisinin-piperaquine (DHP) to be safe and highly effective (failure rates <10%) in treating uncomplicated malaria from both species in non-pregnant patients [12]. Preliminary data on the use of piperaquine from animal reproductive toxicity studies are reassuring [13], [14], [15], but few studies have specifically documented the safety and efficacy of DHP in pregnant women with uncomplicated *P. falciparum* malaria [16], [17].

In view of the high level of multidrug resistant malaria and malaria attributable morbidity and mortality, the local Ministry of Health in southern Papua changed antimalarial policy for uncomplicated malaria to DHP in March 2006, for all patients over 5 kg in weight including pregnant women with uncomplicated malaria in the second and third trimesters. At the same time the policy for the initial treatment of pregnant women with severe malaria in the second and third trimester of pregnancy was changed to intravenous artesunate followed with DHP. The aim of the current study was to document the exposure of pregnant women with malaria to DHP in order to assess their safety in both mother and baby and impact on pregnancy outcome.

## Methods

### Study site

The study was carried out at Rumah Sakit Mitra Masyarakat (RSMM) hospital, Timika, (Papua, Indonesia) a hospital servicing a population of 200,000 people. Malaria transmission is unstable with an annual incidence of 876 per 1000 person years, divided 61:39 between *P. falciparum* and *P. vivax* infections [18]. Clinical drug trials in this region have shown more than 50% of patients with either *P. falciparum* or *P. vivax* fail treatment following chloroquine monotherapy, CQ plus SP or unsupervised quinine [11].

### Study population

The maternal mortality ratio in this region is 1,145/100,000 live births with the infant mortality rate reaching 68 per 1,000 live births [19]. Each year there are approximately 3,000 pregnant women in Timika, of whom less than 40% attend antenatal care clinic and only half deliver at the RSMM hospital [20]. As well as local lowland Papuans, other ethnic groups are present in this region attracted by a local mine.

### Data collection

From April 2004 to June 2009, all pregnant women and newborn infants admitted to the maternity ward at RSMM were screened for malaria and, following written informed consent, data were gathered prospectively using a systematic questionnaire as described previously [9]. Maternal venous blood (5 ml) was taken on admission, for complete blood count and blood film examination by accredited research microscopists. Parasite counts were determined from the number of parasites per 200 white blood cells (WBC) on Giemsa-stained thick films and considered negative after review of 200 high-power fields. A thin smear was examined to confirm parasite species and quantify parasitemia if greater than 200 per 200 WBC on the thick film. Haemoglobin concentration was determined by electronic counter (Coulter JT™, USA).

All pregnant women admitted to hospital were screen for malaria and any found to have a peripheral asexual parasitemia were offered antimalarial treatment, irrespective of clinical signs, species of infection or the degree of parasitaemia.

Severe malaria was defined according to the 2003 WHO criteria {WHO, 2003 #1242}.

Maternal anemia was categorized as severe if the hemoglobin concentration was less than 7 g/dl [21]. Neonatal adverse outcomes (low birth weight, preterm delivery and perinatal deaths) were defined according to WHO criteria [22]. On admission to hospital, fever was diagnosed if women gave a history of fever within the preceding 24 hours or had a documented axillary temperature greater than 37.5°C. Women who gave a history of a febrile illness during this pregnancy were regarded as having a history of possible malaria.

Antimalarial treatment for uncomplicated malaria was administered according to hospital policy, which until March 2006 was quinine plus clindamycin for patients with falciparum malaria and chloroquine plus sulfadoxine-pyrimethamine for women with non-falciparum malaria. Treatment was administered by the ward nurse during in patient stay but was unsupervised if the patient was discharged from hospital prior to completing treatment. Women with manifestations of severe malaria or judged as being at risk by the attending clinician were treated with intravenous quinine. In March 2006 local therapeutic guidelines for both uncomplicated and severe malaria were revised. Pregnant women with uncomplicated malaria due to any species of Plasmodium in the second and third trimester of pregnancy were treated with oral dihydroartemisinin-piperaquine. Quinine and clindamycin remained the treatment of choice in the first trimester. Dihydroartemisinin-piperaquine (DHP) was produced by Holley Pharmaceutical Co, PRC, the fixed dose containing 40 mg dihydroartemisinin and 320 mg piperaquine (Artekin®) administered according to body weight with a target dose of 2–4 mg/kg of dihydroartemisinin and 16–32 mg/kg of piperaquine, once a day for three days. During periods in which the supply of DHP was interrupted, an alternative ACT (amodiaquine plus artesunate) was substituted. Women with severe manifestations of malaria as defined by the WHO guidelines {WHO, 2003 #1242}, were treated with intravenous artesunate until they could tolerate oral treatment with oral DHP (2^nd^ and 3^rd^ trimesters) or quinine (1^st^ trimester).

The attending physician and a research clinician prospectively reviewed all maternity ward admissions and newborns. A systematic interview and review of the clinical notes was used to obtain data on maternal characteristics (maternal age, ethnic groups, parity), clinical condition (fever and signs of severity as defined by WHO guidelines [8]), history of febrile illness and malaria treatment received during the current pregnancy. A trained research nurse performed newborn physical examination to assess clinical signs and gestational age (New Ballard Score) [23].

### Statistical analysis

Data were entered into EpiData 3.02 software (EpiData Association, Odense, Denmark) and statistical analysis done using SPSS vs17.0 (SPSS Inc, Chicago, Illinois). Since pregnant women could not be randomized to different treatments, comparisons were made between maternal outcome in those exposed to DHA-piperaquine and compared with women treated other antimalarials, before and after controlling for baseline confounding factors. Antimalarial exposure during pregnancy was evaluated according to two analyses. The first analysis included women presenting with peripheral parasitaemia at the time of admission. In these individuals malaria itself is an important cause of adverse outcomes such as abortion, premature delivery and intra-uterine fetal death. To reduce this confounding effect the risk of adverse events associated with drug treatment excluded women presenting with an impending outcome prior to treatment. Women who had no adverse outcome during an acute admission for malaria were defined as being discharged with an ongoing pregnancy. The second analysis included all women with a known outcome of pregnancy to assess the risk of adversity associated with exposure to antimalarial drugs over the prior antenatal period. The analyses of drug exposure were restricted to the six main treatments recommended for uncomplicated malaria (chloroquine +/− sufadoxine pyrimethamine, oral quinine and oral DHA-piperaquine) and severe malaria (intravenous quinine, intravenous artesunate and intravenous artesunate plus oral DHA-piperauine).

Categorical data were compared by χ^2^ with Yates' correction or by Fisher's exact test. Normally distributed continuous data were compared by Student's t test or one-way analysis of variance if normally distributed and by Mann-Whitney U test or the Wilcoxon signed rank test if non normally distributed. Survival analysis with the Kaplan Meier method was used to examine the effect of malaria treatment history during pregnancy on the cumulative risk of malaria at delivery. Multiple logistic regression was used to determine the adjusted odds ratios (AOR) of risk factors for adverse pregnancy outcomes as defined in previous analysis from the same location ; these included maternal age, ethnic groups, parity, fever and severity of malaria [9]. In all multivariable models, known risk factors were entered into the model after stratifying by year of exposure.

### Ethical approval

Written informed consent was gathered from all patients or in the case of minors their guardians. Ethical approval for the study was obtained from the ethics committees of the National Institute of Health Research and Development, Ministry of Health, Indonesia (KS.02.01.2.1.3431) and Menzies School of Health Research, Darwin, Australia (04/47).

## Results

Between April 2004 and June 2009, 6519 pregnant women were admitted to the maternity ward and enrolled in the study. Of these women 5% (322) were admitted more than once. The reason for admission was primarily for delivery (4963/6508, 76%) with the remaining women admitted for malaria (728, 11%) and impending adverse outcomes (817, 12.5%). In 11 patients the reason for admission was not documented.

Of the 6475 (99%) pregnant women screened for malaria, 1682 (26%) had a peripheral parasitaemia, 1050 (16.2%) with *P. falciparum*, 456 (7%) with *P. vivax* 62 (3.7%) with *P. malariae*, one with *P. ovale* and 113 (1.7%) with mixed infections (mostly *P. falciparum* and *P. vivax*). In total 893 (53%) women with malaria were symptomatic (fever or history of fever), with severe disease recorded in 106 (6.3%). Of those with severe disease, 73% (77) had *P. falciparum* infections, with the remaining equally distributed between *P. vivax (10), P. malariae* (9), and mixed infections (10). The most common manifestation of severity was severe anemia (hemoglobin <7 g/dl), present in 81 (76%) women. The maternal mortality ratio was 324 per 100,000 live births (11/3,398 live births) overall and 298 per 100,000 live births (5/1680) in women with malaria. Three of these malaria-associated maternal deaths were infected with *P. falciparum* and the other two with *P. vivax*.

### Exposure to antimalarial medications in the current episode of malaria

**Figure 1:**
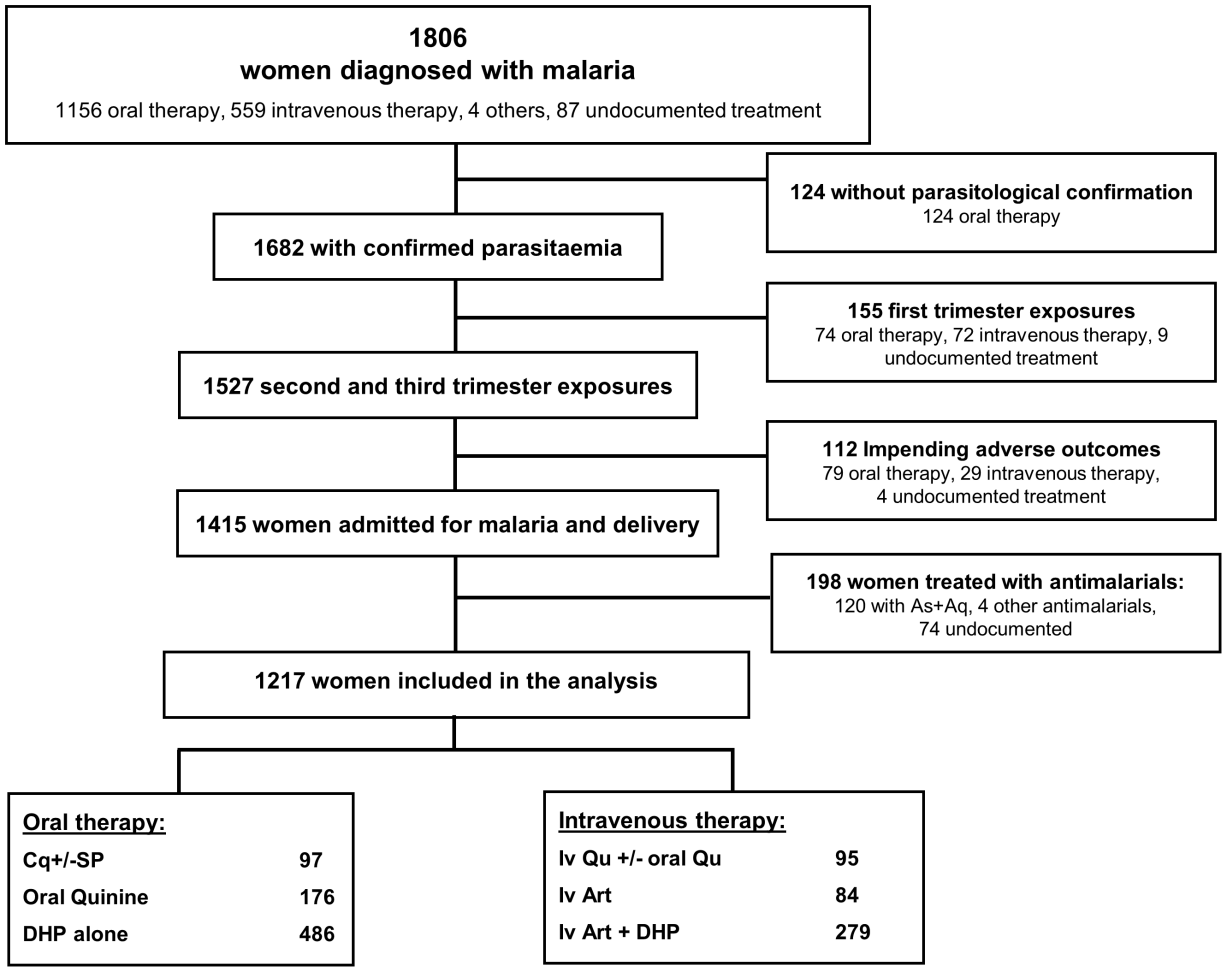
Study Profile: Acute Malaria Treatment.

A total of 1217 women in the second and third trimester had detectable peripheral parasitemia, presented to hospital without impending adverse outcomes; **Figure 1**. Of these 97 (7.9%) were treated with CQ+/−SP, 271 (22%) with oral/IV Quinine, 486 (40%) with DHP alone and 363 (30%) with intravenous artesunate with or without DHP.

Of the 525 mothers admitted for the treatment of malaria, 93% (384/413) of those receiving an arteminisin based treatment (oral DHP or intravenous artesunate) were discharged with an ongoing pregnancy compared to 84% (96/114) of those receiving a non artemisinin treatment (Odds Ratio = 2.48 [1.26–4.86]); p = 0.006. The difference was most apparent in the risk of neonatal death in patients receiving oral treatment in whom none (0/107) of their babies of mothers treated with DHP had early neonatal death, compared 6.5% (3/46) of babies born of mothers treated with oral quinine (p = 0.026, **Table 1**); this difference remained after controlling for baseline confounding factors (maternal age, ethnic groups, parity, fever and severity status): Adjusted Odds Ratio (AOR) = 0.2 [95%CI, 0.49–0.87], p = 0.032.

**Table 1:** Pregnancy outcomes in women with acute malaria (2^nd^ and 3^rd^ trimesters of pregnancy).

Pregnancy Outcome	CQ+/−SP	Qu	DHP alone	p value	IV Qu +/− Oral Qu	IV Art + DHP	IV Art alone	p value	p value (oral vs IV)
	n = 97	n = 176	n = 485§		n = 95	n = 279§	n = 83		
**Admitted for Delivery (n = 690)**									
Live births	99% (93/94)	96% (125/130)	97 (366/378)	0.455	90% (27/30)	96% (43/45)	92% (12/13)	0.640	0.110
Still births	0% (0/94)	0.8% (1/130)	1.3% (5/378)	0.491	3.3% (1/30)	4.4% (2/45)	0% (0/13)	0.739	0.078
Early neonatal deaths	1.1% (1/94)	3.1% (4/130)	1.6% (6/378)	0.459	3.3% (1/30)	0% (0/45)	7.7% (1/13)	0.233	0.676
**Admitted for Treatment of Malaria (n = 525)** §									
Ongoing pregnancy	100% (3/3)	83% (38/46)	95% (102/107)	0.029	85% (55/65)	92% (215/234)	96% (67/70)	0.064	0.345
Still births	0% (0/3)	0% (0/46)	0% (0/107)	-	3.1% (2/65)	3.8% (9/234)	2.9% (2/70)	0.904	0.013
Early neonatal deaths	0% (0/3)	6.5% (3/46)	0% (0/107)	0.026	0% (0/65)	0% (0/234)	0% (0/70)	-	0.051
Preterm delivery*	0% (0/3)	2.9% (1/34)	1.2% (1/86)	0.766	0% (0/54)	2% (4/203)	0% (0/64)	0.308	0.707

**Notes:**

**CQ** = Chloroquine; **SP** = Sulphadoxine-Pyrimethamine; **DHP** = Dihydroartemisinin piperaquine; **Qu** = Quinine; **Art** = Artesunate.

**CQ+/−SP** = chloroquine with or without Sulphadoxine-Pyrimethamine;

**IV Qu +/− Oral Qu** = Intravenous Quinine with our without subsequent oral quinine.

In women presenting before 37 weeks gestation;

undocumented pregnancy outcomes in two women (one treated with DHP and the other with IVArt).

An additional 124 women were diagnosed and treated for malaria but subsequently found to be aparasitaemic. The 93 women presenting without an impending adverse outcome (2 Cq/SP, 13 Quinine and 78 DHP) had all been admitted for delivery and delivered live babies.

Of the 332 women admitted in the first trimester, 152 (45.8%) were found to have a peripheral parasitemia, and 41 of these women had impending miscarriage at the time of presentation. In two cases antimalarial treatment was not documented. Of the remaining 109 women 8 (7.3%) miscarried, the risk being 2.6% (1/38) in those receiving oral quinine compared to 62.5% (5/8) in those treated with an oral DHP; p<0.001. None of the 60 women receiving intravenous therapy miscarried (50 with intravenous quinine, 5 with intravenous artesunate plus DHP and 5 with intravenous artesunate alone).

### History of prior antimalarial medication

**Figure 2:**
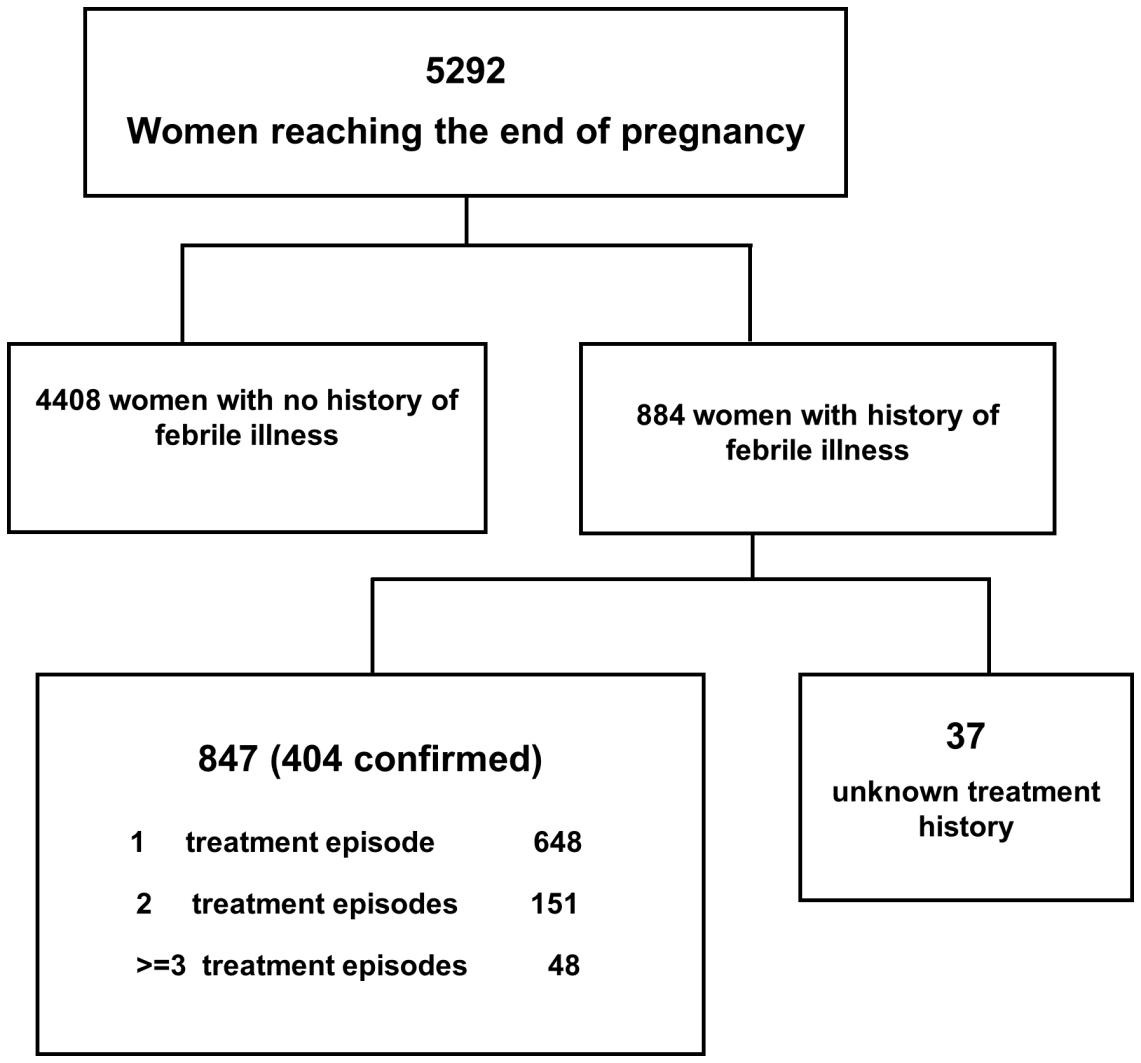
Study Profile: Pregnant Women Delivering with Prior Febrile Illness.

The outcome of pregnancy could be determined in 5292 (81.7%) women, of whom 884 (16.7%) reported a history of febrile illness during the pregnancy. Nearly all (847, 96%) of these women were able to state their prior malaria treatment, and in 404 (47.7%) cases the diagnosis and treatment could be confirmed from hospital records with >95% concordance (**Figure 2**). Compared to women without any history of febrile illness, those with prior febrile illness were more likely to be younger, Papuan and primigravidae (**Table 2**).

**Table 2:** Baseline characteristic of pregnant women delivering with prior history of last antimalarial exposure.

Characteristic	No history of malaria (n = 4408)	CQ±SP (n = 24)	Oral Qu (n = 347)	DHP alone (n = 336)	IV Art +/− DHP (n = 77)
**Age, mean year (95%CI of mean)**	25.9 (25.8–26.1)^a^	23.7 (21.3–26.2)	25.4 (24.8–26.0)	24.8 (24.1–25.4)	24.6 (23.3–25.9)
**Papuan Ethnic groups**	64.7% (2846/4399)^a^	75% (18/24)	61.6% (213/346)	83% (279/336)^c^	71.4% (55/77)
**Primigravidae**	31% (1365/4404)^b^	45.8% (11/24)	37% (129/347)	32.4% (109/336)	39% (30/77)
**Gestation of pregnancy, median week (range)**	39 (19–44)^a^	39 (34–40)	39 (20–43)	38 (24–44)	38 (21–41)^d^
**Febrile at delivery**	5.2% (231/4406)^a^	33.3% (8/24)	28% (97/347)	10.7% (36/336)^c^^,^ e	9% (7/77)^c^^,^ e
**Number of febrile episodes during pregnancy, median no. (range)**	-	1 (1–2)	1 (1–8)	1 (1–7)^e^	1 (1–4)^e^
**Time from last exposure to delivery, median weeks (range)**	-	16 (1–35)	12 (1–39)	10 (1–29)^c^^,^ e	10 (1–23)^d^

**Notes:** Compared with prior history of last antimalarial exposure:

^a^ p<0.001,

^b^ p<0.05; Compared with Oral Quinine:

^c^ p≤0.001,

^d^ p<0.05; Compared with CQ+/−SP:

^e^ p<0.05.

Prior drug exposure during the same pregnancy for peripheral parasitaemia, ranged from 1 to 8 episodes, although the majority of women had a single antimalarial exposure (76%, 648/847); 122 women had multiple exposures with the same drug and 77 received different drugs, which were mostly quinine treatment followed by DHP (61%, 47/77). The potential effect of drug exposure to pregnancy outcome was assessed according to the last anti-malarial exposure controlling for multiple exposure. The last treatment of malaria was documented in 784 women of whom 102 (13%) were exposed in the first trimester, 238 (30%) in the second trimester and 441 (56%) in the third trimester of pregnancy. For three women the timing of exposure was unknown. Women with prior exposure to DHP were more likely to have received treatment closer to delivery and to be afebrile on admission compared to those treated with past CQ and oral Quinine (**Table 2**). There were no other significant differences in baseline characteristics between women exposed to different antimalarial regimens.

**Figure 3:**
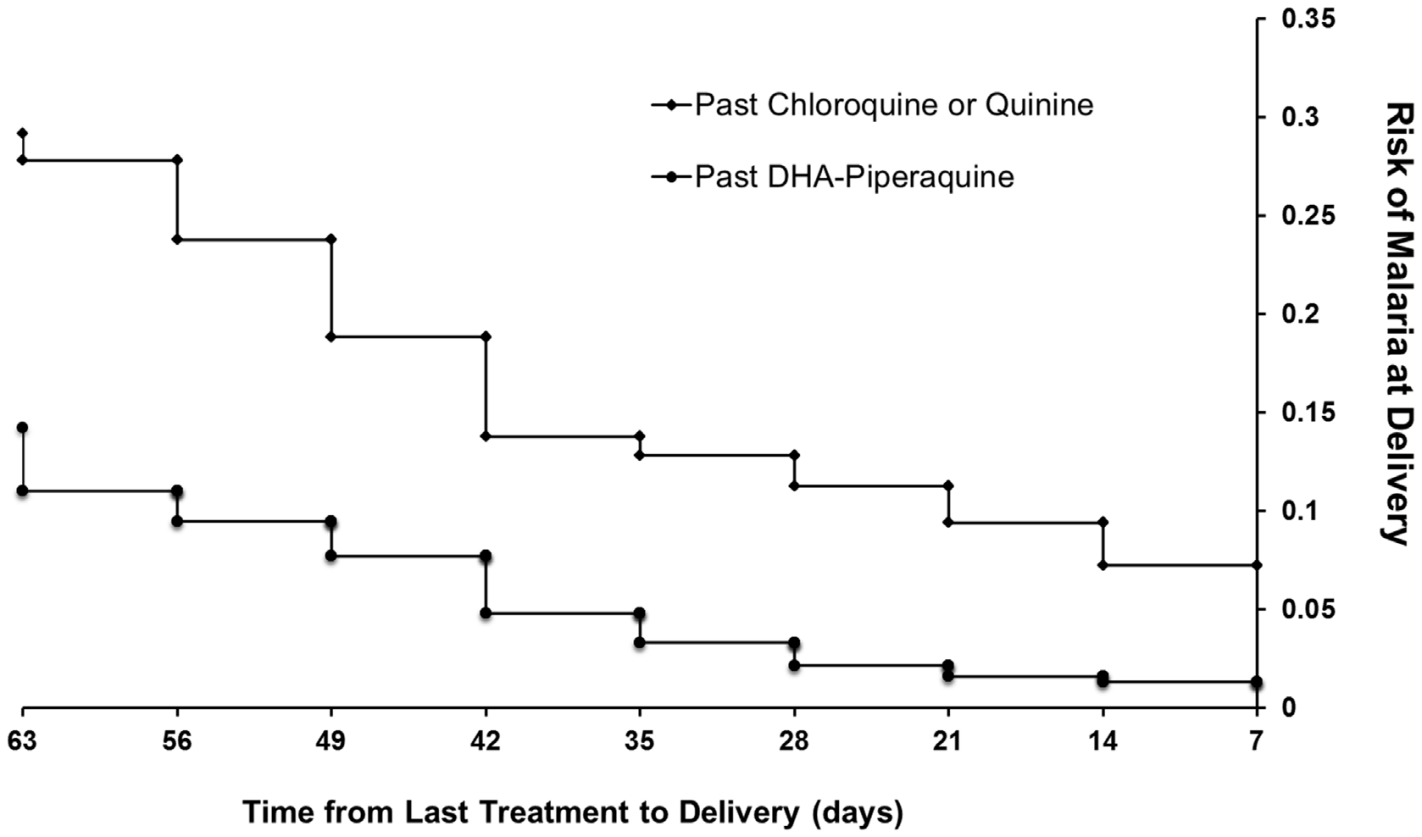
The proportion of women with peripheral parasitaemia at the time of delivery according to the timing of prior antimalarial treatment.

Women reporting prior febrile illness were at significantly greater risk of parasitemia at delivery compared to those with no febrile history (OR = 3.65 [95%CI, 3.07–4.34], p<0.001). The risk of parasitemia at delivery was 26% (86/336) in women with prior DHP treatment compared to 48% (178/370) of those previously treated with oral quinine or CQ+/−SP; OR = 0.37 (95%CI 0.27–0.52), p<0.001, **Table 3** and **figure 3**. This difference remained significant in the multivariate model (AOR = 0.48 [95%CI, 0.35–0.66], p<0.001). The lower risk of parasitemia at delivery following DHP was apparent for both *P. falciparum* (OR = 0.34 [95%CI, 0.24–0.49], p<0.001) and *P. vivax* (OR = 0.6 [95%CI, 0.37–0.96], p = 0.034). The median time from last treatment to representation with malaria was 11 weeks [range: 1–27] following DHP treatment compared to 8 weeks [range: 1–37], following non ACT therapy, p = 0.05.

**Table 3:** Adverse outcomes associated with prior last history of antimalarial exposure.

Adverse Outcomes	No exposure and history of malaria illness (n = 4408)	CQ+/− SP (n = 24)	Oral Qu (n = 347)	IV Art +/− DHP (n = 77)	DHP alone (n = 336)	P value^a^	P value^b^
**Maternal malaria at delivery**	13.6% (596/4378)	56.5% (13/23)	47.6% (165/347)	28.6% (22/77)	25.6% (86/336)	<0.001	<0.001
**Maternal severe anemia**	8% (344/4291)	17.4% (4/23)	12% (41/341)	11% (8/72)	12.2% (39/319)	<0.001	0.880
**Low birth weight**	14.7% (642/4382)	25% (6/24)	20.4% (70/343)	14.3% (11/77)	18% (60/333)	<0.001	0.507
**Mean birth weight, 95%CI of mean**	3028 (3011–3045)	2726 (2478–2974)	2884 (2819–2950)	2859 (2759–2958)	2905 (2844–2967)	<0.001	0.698
**Preterm delivery**	10.8% (450/4158)	21.7% (5/23)	12.9% (41/318)	18.7% (14/75)	16.9% (55/326)	<0.001	0.337
**Perinatal death**	4% (177/4408)	0% (0/24)	6.6% (23/347)	2.6% (2/77)	3.3% (11/336)	0.510	0.091
**Still births**	3% (134/4408)	0% (0/24)	4.6% (16/347)	2.6% (2/77)	2.7% (9/336)	0.625	0.386
**Early neonatal deaths**	1% (43/4408)	0% (0/24)	2% (7/347)	0% (0/77)	0.6% (2/336)	0.800	0.222
**Congenital malaria**	0.6% (20/3409)	5% (1/20)	4.6% (14/302)	0% (0/75)	0.3% (1/326)	<0.001	0.001

^a^ P value refers to comparison of patients with no history of malaria compared to those with any history of malaria combining all treatments.

^b^ P value refers to difference between prior treatments.

Prior antimalarial exposure was a significant risk factor for adverse pregnancy outcomes compared to those without any history of malaria during pregnancy (**Table 3**). The risk of perinatal death was 6.6% (23/347) in babies born to women with prior quinine exposure compared to 3.3% (11/336) in those born of mothers previously treated with oral DHP, and an adjusted Odds Ratio of 3.17 [95%CI: 1.17–8.60] after controlling for confounding factors; p = 0.023 (**Table 3** **and** **4**). There was also a significant difference in the risk of congenital malaria which was greater in women with a history of prior quinine exposure compared to those exposed to DHP (OR = 15.8 [95%CI, 2.1–121], p = 0.001) (**Table 3**), although this was not apparent in the multivariate analysis (**Table 4**).

**Table 4:** Predictors for Adverse Outcomes.

Predictors	Maternal Malaria at delivery	Maternal severe anemia	Low Birth weight	Preterm delivery	Perinatal death	Congenital malaria
**Maternal Malaria at delivery**	-	3.03 (1.83–5.01), p<0.001	2.08 (1.34–3.22), p = 0.001	1.74 (1.06–2.87), p = 0.029	2.43 (1.10–5.39), p = 0.029	3.25 (0.56–19.06), p = 0.191
**Maternal severe anemia**	3.05 (1.83–5.07), p<0.001	-	2.61 (1.57–4.33), p<0.001	2.01 (1.13–3.55), p = 0.018	1.22 (0.47–3.15), p = 0.684	1.29 (0.32–5.16), p = 0.721
**Fever/History of fever**	9.04 (5.62–14.53), p<0.001	0.78 (0.44–1.39), p = 0.404	1.38 (0.83–2.29), p = 0.208	2.45 (1.39–4.29), p = 0.002	0.93 (0.38–2.28), p = 0.881	1.48 (0.47–4.68), p = 0.508
**Primigravidae**	1.17 (0.78–1.76), p = 0.448	1.05 (0.62–1.78), p = 0.868	2.56 (1.63–4.02), p<0.001	2.16 (1.31–3.54), p = 0.002	1.87 (0.83–4.23), p = 0.132	0.52 (0.14–1.97), p = 0.336
**Papuan Ethnicity**	1.23 (0.82–1.85), p = 0.322	2.42 (1.25–4.68), p = 0.009	0.99 (0.62–1.59), p = 0.975	0.92 (0.55–1.56), p = 0.757	2.66 (0.99–7.12), p = 0.051	0.68 (0.19–2.48), p = 0.560
**Maternal age ≤18 y.o**	0.97 (0.93–1.00), p = 0.065	0.97 (0.92–1.01), p = 0.152	1.0 (0.96–1.04), p = 0.910	0.99 (0.95–1.04), p = 0.856	1.02 (0.95–1.09), p = 0.567	1.04 (0.95–1.14), p = 0.421
**Prior Treatment Exposures**						
**CQ+/− SP**	1.52 (0.51–4.49), p = 0.450	0.87 (0.24–3.18), p = 0.831	1.03 (0.33–3.16), p = 0.966	1.10 (0.33–3.71), p = 0.874	-	0.87 (0.03–23.44), p = 0.933
**Qu+/−IV Qu**	1.56 (0.97–2.50), p = 0.068	0.75 (0.39–1.48), p = 0.411	0.90 (0.52–1.55), p = 0.707	0.59 (0.32–1.1), p = 0.097	3.17 (1.17–8.60), p = 0.023	1.16 (0.09–14.64), p = 0.909
**IV Art+/−DHP**	1.13 (0.61–2.12), p = 0.690	0.89 (0.39–2.07), p = 0.798	0.69 (0.32–1.45), p = 0.325	1.02 (0.49–2.10), p = 0.967	0.94 (0.19–4.45), p = 0.938	-
**DHP alone**	1	1	1	1	1	1

Footnote: Adjusted Odds Ratio (95% Confidence intervals) are presented. In all multivariate models cofactors were entered into the model after stratifying by year of exposure.

## Discussion

In Papua Indonesia both *P. falciparum* and *P. vivax* infections occur in approximately 20% of pregnant women and, even when asymptomatic, are associated with an increased risk of maternal anemia, still birth, prematurity, low birth weight and perinatal death [9]. Treatment options are limited since high grade multidrug resistance has emerged in both *P. falciparum* and *P. vivax* to chloroquine, amodiaquine, and sulfadoxine-pyrimethamine [11], [24], [25]. Antimalarial resistance is described less frequently with quinine, however the prolonged thrice daily treatment regimen is associated with poor adherence and unacceptable effectiveness, which is likely to contribute significantly to detrimental outcomes on both mother and baby. In areas where adherence to a complete course of treatment cannot be assured and multidrug resistance is rising, the use of these conventional treatments demands careful scrutiny. The necessary revision of the local district antimalarial policy in Timika in March 2006, saw the introduction of dihydroartemisinin-piperaquine (DHP) for the treatment of uncomplicated malaria due to any species, including the second and third trimesters of pregnancy. Our study allowed us to review 1217 pregnant women treated with antimalarial drugs including more than 760 women with antenatal exposure to DHP.

In the second and third trimesters mothers the outcomes following treatment with oral DHP were at least as good as those for women treated with oral quinine. Infact in those presenting with a febrile illness, DHP resulted in a 2.5 fold greater likelihood of being discharged from hospital with an ongoing pregnancy compared to women receiving oral quinine. The policy switch from quinine to DHP for uncomplicated malaria in March 2006 was associated with a significant reduction in the risk of early neonatal death from 6.5% (3/46) to zero (0/107). Significant differences were also apparent in pregnant women with a history of prior antimalarial exposure. Compared to women previously treated with chloroquine or quinine, those treated with DHP had a lower risk of malaria at delivery (OR = 0.37), an effect that extended for treatment up to 9 weeks prior to delivery (**Figure 3**). This differential effect may reflect a number of factors including the proven efficacy of DHP against the drug resistant isolates of both *P. falciparum* and *P. vivax* prevalent in this region, as well as the prolonged post treatment prophylaxis afforded by piperaquine's long terminal elimination half life (∼28 days) apparent in non pregnant [26] and pregnant women [27]. Although a 7 day regimen of quinine given three times per days may have adequate efficacy when supervised, it reliably causes unpleasant side effects and patients rarely adhere to a complete treatment course. The poor adherence to quinine has major implications for high rates of recrudescent infection and its short elimination half life provides minimal post treatment prophylaxis against new and relapsing infections. Our results suggest the increased risk of recurrent parasitemia following quinine was associated with a significantly greater risk of perinatal mortality and congenital malaria.

In the first trimester the risk of abortion was over 60% in women receiving an ACT (7/11) compared to only 1% (1/38) in women treated with quinine. Interestingly none of the 10 women receiving IV artesunate, either with and without DHP, miscarried. The markedly higher rates of abortion in women with first trimester exposure to DHP is of concern, and although this may signify drug toxicity, it may also reflect intrinsic biases within our non-comparative observational study. The abortion risk of women treated with quinine in our study was significantly lower than that described in women on the Thai-Burmese border (25%) [28] suggesting a possible systematic bias in prescribing alternative medication in this risk group. Anecdotal evidence indicates that in our study prescription of oral DHP alone in the first trimester was reserved for those women who were more unwell, even though this was not recommended in local protocols and this may explain the poor outcome.

There are a number of limitations to our study. Following policy change in 2006, it was not deemed acceptable to randomize women to treatments known to be partially effective, hence our study is reliant on historical comparison and all the confounding factors inherent within that. Although multivariable analysis was applied to control for confounders, other unrecorded factors will not have been included and a degree of residual confounding is likely to have contributed for some of the differences in outcomes observed. However our analysis represents one of the largest observational studies to date of the use of DHP in pregnancy and provides reassuring data that in the second and third trimesters its use is associated with outcomes at least as good that following quinine. Furthermore the simple three day treatment regimen has pragmatic implications for adherence properties and when combined with the slow elimination of piperaquine it resulted in a significant reduction in recurrent parasitaemia with associated benefits for both maternal and neonatal outcomes. As such these observations provide reassuring data and an incentive for further comparative studies of DHP in pregnancy, including a potential role for DHP as a novel intermittent presumptive therapy [29].

Another limitation was the use of information of prior antimalarial exposure derived from questioning of the mother and thus vulnerability to recall bias. We used a systematic interview method to obtain information on the timing of exposures (approximate pregnancy gestational age in months) and treatment received (shape and colour of the tablets). Our experience has shown that women in this region are familiar with the various available antimalarial drugs and this was confirmed by very high concordance between the interview results and the actual drug received in women whose medical record were available. Reassuringly in a sensitivity analysis in which the data were restricted to the women in whom documentation could be confirmed from hospital records, the observations of adverse outcomes were similar.

Pregnant women were not routinely followed up following antimalarial exposure and documentation of birth outcome could only be made in 17% of pregnant women exposed to antimalarials on hospital admission and discharged with ongoing pregnancy. We can not discount the possibility of women exposed to DHP having adverse outcomes away from hospital, however it is likely to be similar for DHP as for chloroquine and quinine. The comparison between treatments therefore provides a useful indication of relative adverse outcomes which in the second and third trimesters were in favour of DHP. Finally the lack of prospective follow up limited our ability to define precisely the efficacy of treatment regimens, although recurrence at representation to hospital was significantly lower in patients receiving DHP compared to chloroquine.

In conclusion, our results show that the early diagnosis and prompt treatment with DHP in the second and third trimesters of pregnancy reduces recurrent malaria and is associated with as fall in perinatal death and congenital malaria. Further prospective randomised controlled trials of DHP in pregnant women are needed to confirm these findings and complementary pharmacokinetic studies to ensure the optimal deployment of this important antimalarial in antenatal practice. The increased rates of abortion in women with exposure in the first trimester is of concern, and although this may reflect bias within our observational study, the use of DHP in the first trimester should continue to be avoided until further safety information is available.

## References

[1] Desai M, ter Kuile FO, Nosten F, McGready R, Asamoa K, et al (2007) Epidemiology and burden of malaria in pregnancy. Lancet Infect Dis 7: 93–104. 17251080 10.1016/S1473-3099(07)70021-X

[2] Brabin BJ, Hakimi M, Pelletier D (2001) An analysis of anemia and pregnancy-related maternal mortality. J Nutr 131: 604S–614S discussion 614S–615S. 11160593 10.1093/jn/131.2.604S

[3] WHO/AFRO (2004) A strategic framework for malaria prevention and control during pregnancy in the African region. World Health Organization Regional Office for Africa

[4] Nosten F, McGready R, Mutabingwa T (2007) Case management of malaria in pregnancy. Lancet Infect Dis 7: 118–125. 17251082 10.1016/S1473-3099(07)70023-3

[5] Rijken MJ, McGready R, Boel ME, Poespoprodjo R, Singh N, et al (2012) Malaria in pregnancy in the Asia-Pacific region. Lancet Infect Dis 12: 75–88. 22192132 10.1016/S1473-3099(11)70315-2

[6] World Health Organisation (2010) Guidelines for the treatment of malaria.

[7] McGready R, Lee SJ, Wiladphaingern J, Ashley EA, Rijken MJ, et al (2012) Adverse effects of falciparum and vivax malaria and the safety of antimalarial treatment in early pregnancy: a population-based study. Lancet Infect Dis 12: 388–396. 22169409 10.1016/S1473-3099(11)70339-5 PMC3346948

[8] WHO (2010) Guidelines for the treatment of malaria. Geneva. pp. 16,28,29,38,39.

[9] Poespoprodjo JR, Fobia W, Kenangalem E, Lampah DA, Warikar N, et al (2008) Adverse pregnancy outcomes in an area where multidrug-resistant plasmodium vivax and Plasmodium falciparum infections are endemic. Clin Infect Dis 46: 1374–1381. 18419439 10.1086/586743 PMC2875100

[10] Nosten F, McGready R, d'Alessandro U, Bonell A, Verhoeff F, et al (2006) Antimalarial drugs in pregnancy: a review. Curr Drug Saf 1: 1–15. 18690910 10.2174/157488606775252584

[11] Ratcliff A, Siswantoro H, Kenangalem E, Wuwung M, Brockman A, et al (2007) Therapeutic response of multidrug-resistant Plasmodium falciparum and P. vivax to chloroquine and sulfadoxine-pyrimethamine in southern Papua, Indonesia. Trans R Soc Trop Med Hyg 101: 351–359. 17028048 10.1016/j.trstmh.2006.06.008 PMC2080856

[12] Ratcliff A, Siswantoro H, Kenangalem E, Maristela R, Wuwung RM, et al (2007) Two fixed-dose artemisinin combinations for drug-resistant falciparum and vivax malaria in Papua, Indonesia: an open-label randomised comparison. Lancet 369: 757–765. 17336652 10.1016/S0140-6736(07)60160-3 PMC2532500

[13] Clark RL, White TE, S AC, Gaunt I, Winstanley P, et al (2004) Developmental toxicity of artesunate and an artesunate combination in the rat and rabbit. Birth Defects Res B Dev Reprod Toxicol 71: 380–394. 15617018 10.1002/bdrb.20027

[14] Clark RL, Arima A, Makori N, Nakata Y, Bernard F, et al (2008) Artesunate: developmental toxicity and toxicokinetics in monkeys. Birth Defects Res B Dev Reprod Toxicol 83: 418–434. 18702116 10.1002/bdrb.20163

[15] Batty KT, Moore BR, Stirling V, Ilett KF, Page-Sharp M, et al (2010) Investigation of reproductive toxicity of piperaquine in mice. Reprod Toxicol 29: 206–213. 19892009 10.1016/j.reprotox.2009.10.013

[16] Rijken MJ, McGready R, Boel ME, Barends M, Proux S, et al (2008) Dihydroartemisinin-piperaquine rescue treatment of multidrug-resistant Plasmodium falciparum malaria in pregnancy: a preliminary report. Am J Trop Med Hyg 78: 543–545. 18385345

[17] Tarning J, Rijken MJ, McGready R, Phyo AP, Hanpithakpong W, et al (2012) Population pharmacokinetics of dihydroartemisinin and piperaquine in pregnant and nonpregnant women with uncomplicated malaria. Antimicrob Agents Chemother 56: 1997–2007. 22252822 10.1128/AAC.05756-11 PMC3318332

[18] Karyana M, Burdarm L, Yeung S, Kenangalem E, Wariker N, et al (2008) Malaria morbidity in Papua Indonesia, an area with multidrug resistant Plasmodium vivax and Plasmodium falciparum. Malar J 7: 148. 18673572 10.1186/1475-2875-7-148 PMC2518158

[19] Hidayat M (2001) Rapid Survey on Maternal Mortality in Papua Province. Provincial Health Office (PHO), Papua Indonesia.

[20] Mimika DHO (2005) Mimika District Health Office Annual Statistics.

[21] Shulman CE, Graham WJ, Jilo H, Lowe BS, New L, et al (1996) Malaria is an important cause of anaemia in primigravidae: evidence from a district hospital in coastal Kenya. Trans R Soc Trop Med Hyg 90: 535–539. 8944266 10.1016/s0035-9203(96)90312-0

[22] WHO (1996) Catalogue of health indicators: a selection of important health indicators recommended by WHO programmes. Geneva. pp. 20 and 51.

[23] Ballard JL, Khoury JC, Wedig K, Wang L, Eilers-Walsman BL, et al (1991) New Ballard Score, expanded to include extremely premature infants. J Pediatr 119: 417–423. 1880657 10.1016/s0022-3476(05)82056-6

[24] Hasugian AR, Purba HL, Kenangalem E, Wuwung RM, Ebsworth EP, et al (2007) Dihydroartemisinin-piperaquine versus artesunate-amodiaquine: superior efficacy and posttreatment prophylaxis against multidrug-resistant Plasmodium falciparum and Plasmodium vivax malaria. Clin Infect Dis 44: 1067–1074. 17366451 10.1086/512677 PMC2532501

[25] Hasugian AR, Tjitra E, Ratcliff A, Siswantoro H, Kenangalem E, et al (2009) In vivo and in vitro efficacy of amodiaquine monotherapy for treatment of infection by chloroquine-resistant Plasmodium vivax. Antimicrob Agents Chemother 53: 1094–1099. 19104023 10.1128/AAC.01511-08 PMC2650584

[26] Hung TY, Davis TM, Ilett KF, Karunajeewa H, Hewitt S, et al (2004) Population pharmacokinetics of piperaquine in adults and children with uncomplicated falciparum or vivax malaria. Br J Clin Pharmacol 57: 253–262. 14998421 10.1046/j.1365-2125.2003.02004.x PMC1884452

[27] Adam I, Tarning J, Lindegardh N, Mahgoub H, McGready R, et al (2012) Pharmacokinetics of piperaquine in pregnant women in Sudan with uncomplicated Plasmodium falciparum malaria. Am J Trop Med Hyg 87: 35–40. 22764289 10.4269/ajtmh.2012.11-0410 PMC3391055

[28] McGready R, Thwai KL, Cho T, Samuel, Looareesuwan S, et al (2002) The effects of quinine and chloroquine antimalarial treatments in the first trimester of pregnancy. Trans R Soc Trop Med Hyg 96: 180–184. 12055810 10.1016/s0035-9203(02)90297-x

[29] White NJ (2005) Intermittent presumptive treatment for malaria. PLoS Med 2: e3. 15696210 10.1371/journal.pmed.0020003 PMC545196

